# External validation of the Vulnerable Elder’s Survey for predicting mortality and emergency admission in older community-dwelling people: a prospective cohort study

**DOI:** 10.1186/s12877-017-0460-1

**Published:** 2017-03-20

**Authors:** Emma Wallace, Ronald McDowell, Kathleen Bennett, Tom Fahey, Susan M. Smith

**Affiliations:** 1 HRB Centre for Primary Care Research, Royal College of Surgeons in Ireland (RCSI), 123 Stephen’s green, Dublin 2, Ireland; 2 Population Health Sciences Division, Royal College of Surgeons of Ireland (RCSI), Dublin 2, Ireland

## Abstract

**Background:**

Prospective external validation of the Vulnerable Elder’s Survey (VES-13) in primary care remains limited. The aim of this study is to externally validate the VES-13 in predicting mortality and emergency admission in older community-dwelling adults.

**Methods:**

Design: Prospective cohort study with 2 years follow-up (2010–2012). Setting: 15 General Practices (GPs) in the Republic of Ireland. Participants: *n* = 862, aged ≥70 years, community-dwellers Exposure: VES-13 calculated at baseline, where a score of ≥3 denoted high risk. Outcomes: i) Mortality; ii) ≥1 Emergency admission and ≥1 ambulatory care sensitive (ACS) admission over 2 years. Statistical analysis: Descriptive statistics, model discrimination (c-statistic) and sensitivity/specificity.

**Results:**

Of 862 study participants, a total of 246 (38%) were classified as vulnerable at baseline. Fifty-three (6%) died during follow-up and 246 (29%) had an emergency admission. At the VES-13 cut-point of ≥3 denoting high-risk model discrimination was poor for mortality (c-statistic: 0.61 (95% CI 0.54, 0.67), ≥1 emergency admission (c-statistic: 0.59 (95% CI 0.56, 0.63) and ≥1 ACS emergency admission (c-statistic: 0.63 (95% CI 0.60, 0.67).

**Conclusions:**

In this study the VES-13 demonstrated relatively limited predictive accuracy in predicting mortality and emergency admission. External validation studies examining the tool in different health settings and healthier populations are needed and represent an interesting area for future research.

**Electronic supplementary material:**

The online version of this article (doi:10.1186/s12877-017-0460-1) contains supplementary material, which is available to authorized users.

## Background

The terms ‘frailty’ and ‘vulnerability’ are widely used in the gerontology literature [1]. Frailty was originally defined as a condition in older people meeting three or more of the following criteria: i) unintentional weight loss; ii) self-reported exhaustion; iii) slow walking speed; iv) weak grip strength and, v) low physical activity level [2]. The term vulnerability refers to a wider range of older people who are at increased risk of functional decline or death [3]. The Vulnerable Elder’s Survey-13 (VES-13) is a risk prediction tool designed in the United States (US) to predict functional decline and death in older community-dwelling (≥65 years) people over 2 years follow-up [3]. It has good clinical utility, as it is easy to administer and can be used to identify older people at higher risk of poorer health outcomes who can be targeted for community-based interventions. The VES-13 was derived through a methodologically robust process, whereby variables with potential predictive power were identified from the US Medicare database and different models tested for relevant outcomes [3]. The final VES-13 model includes items relating to patient age, self-rated health and the ability to perform specified physical and functional tasks [3]. A score of ≥3 is considered high-risk of experiencing future functional decline or death. It has been successfully validated in several community-based US studies to predict functional decline and death [3–7]. In one such US study (*n* = 649, ≥75 years) for each additional increase in VES-13 point, the odds of functional decline or death increased by almost 40% (odds ratio (OR) 1.37 (95% confidence interval (CI) 1.25, 1.50) and the model’s c-statistic was 0.75 (95% CI 0.71, 0.80) over five-year follow-up [4]. The VES-13 has also been extensively validated to predict various adverse health outcomes in older people with an index diagnosis of cancer [8].

However, validation of the VES-13 outside North America in older people without cancer has been limited [9, 10]. A prospective Dutch study (*n* = 354, aged ≥70 years) with one-year follow-up reported that the VES-13 was significantly associated with functional decline in older persons without cancer (OR 2.83, 95% CI 1.35, 5.95) [9]. One Irish study (*n* = 2,033 aged ≥65 years) examined the cross-sectional association of the VES-13 with healthcare utilisation and reported that people categorised as vulnerable (32%) had higher healthcare use including primary care visits, emergency room (ER) visits and use of hospital services [10]. Predicting emergency admission is of interest, both from a clinical and policy perspective internationally, and the use of risk prediction models to identify high-risk people is increasingly advocated [11, 12]. Adopting the VES-13 to predict emergency admission could have both clinical and policy implications as with an ageing population, examining innovative ways of identifying older people at highest risk is important.

The aim of this study is to examine the VES-13 in predicting mortality and emergency admission in older people. The specific objectives were: 1) To externally validate the VES-13 in predicting mortality in a cohort of older community-dwelling people, and 2) To examine the predictive accuracy of the VES-13 in predicting all-cause emergency admissions and a subset of emergency admissions resulting from ambulatory care sensitive conditions.

## Methods

The STrengthening the Reporting of Observational Studies in Epidemiology (STROBE) guidelines were adhered to in the conduct and reporting of this cohort study [13].

### Study design and study population

This is a two-year prospective cohort study of older patients from general practice (GP) recruited from 15 practices in the Republic of Ireland (2010–2012). This study is part of a larger study examining the prediction of self-reported adverse drug events (ADEs) in older people. A total of 19 general practices affiliated to either the Royal College of Surgeons in Ireland or Trinity College Dublin through undergraduate teaching were approached to take part in the study and 15 practices consented to take part. A proportionate stratified random sampling approach was used to recruit patients at baseline. There were a total of 4,573 patients aged ≥70 years across the 15 practices. Of these a propionate random sample were selected to participate (*n* = 1,764). A total of 1,487 patients remained eligible following application of exclusion criteria and a total of 904 (response rate = 61%) took part in the study at baseline.

Study inclusion criteria were: i) aged ≥70 years on 1^st^ January 2010 and; ii) in receipt of a valid general medical services (GMS) card. Approximately 96% of all people aged ≥70 years in the Republic of Ireland are in receipt of a GMS card which entitles the holder to free public health services (including GP visits) and prescriptions, subject to a maximum co-payment of €25 monthly [14].

As part of the larger study predicting ADEs participants needed to be able to complete an interview regarding their medications and complete a postal questionnaire. As a result the following exclusion criteria were applied: i) Receiving palliative care; ii) Cognitive impairment at the level that would impact their ability to complete the outcome measure (defined as Mini Mental State Examination (MMSE) ≤20); iii) Significant hearing/speech/visual impairment; iv) Currently experiencing a psychotic episode; v) Hospitalised long-term, in a nursing home, homeless or in sheltered accommodation; and, vi) Recent bereavement (within 4 weeks). Each participant’s GP applied the exclusion criteria and determined eligibility for participation at baseline in 2010. Ethical approval for this study was granted by the Royal College of Surgeons in Ireland (RCSI) Human Research Ethics committee and all participants gave informed consent prior to participating. Data was entered into a database and a number of queries developed designed to identify erroneous, missing or duplicate data. Any data errors were checked against the original hard copy questionnaire and corrected. A random sample of 10% of all patient data was double-checked against the original hard copy postal questionnaire

### Exposure of interest: vulnerability

Vulnerability was measured at baseline in 2010 using the Vulnerable Elder’s Survey (VES-13) which includes 13 items relating to patient age, self-rated health, ability to perform six physical tasks (e.g. writing or handing small objects, walking quarter of a mile, lifting) and five items relating to function (e.g. bathing, managing finances, light housework) (See Additional file 1) [3]. The maximum score is 10 points and a cut-off of ≥3 denotes high-risk of experiencing future functional decline. This survey was administered via postal questionnaire to study participants at baseline and again at follow-up in 2012.

### Outcomes

#### Mortality

Details regarding study participants who had died during study follow-up were obtained from the 15 participating general practices.

#### Emergency admission

Emergency admission was defined as ‘unplanned overnight stay in hospital’ [15]. Emergency admission during the 2 years follow up period was recorded by reviewing the study participant’s family practitioner (GP) electronic medical record. The number of emergency admissions, reason for admission, length of hospital stay and date of admission and discharge was recorded. In addition, ambulatory care sensitive (ACS) admissions were identified. These are a subset of all emergency admissions that occur due to select medical conditions (e.g. chronic obstructive pulmonary disease (COPD), congestive heart failure (CHF) and cellulitis) and are considered more amenable to prevention through primary care management [16]. A list of included ACS conditions is provided in Additional file 2. Reasons for emergency admissions were reviewed and those resulting from any one of the ACS conditions listed were coded as an ACS admission.

### Statistical analysis

Baseline descriptive statistics of the cohort are described. Baseline characteristics were assessed by administering a patient self-report questionnaire which was administered at the same time as the VES-13 at baseline and follow-up. In addition each patient’s GP medical record was reviewed and details regarding date of birth, gender and address were extracted. Participant addresses were geocoded to determine which electoral division (ED) or small area they lived in. ED deprivation was based on the Small Area Health Research Unit (SAHRU) national deprivation index, which is similar in design to Carstairs and Townsend indices employed in the United Kingdom, *and* classifies deprivation according to the person’s address [17]. Participants were classified into one of seven social class groups, based on their previous occupation, according to the Irish Central Statistics Office population census classification system as follows: (i) professional workers; (ii) managerial and technical; (iii) non-manual; (iv) skilled manual; (v) semi-skilled; (vi) unskilled; and (vii) all others gainfully occupied and unknown. Patients whose previous occupation was “looking after home or family’ were assigned the social class of their spouse or partner. The seven social class groups were then reclassified into two social classes: the unskilled and those gainfully occupied and unknown were reclassified as unskilled and all other skilled social classes were reclassified as skilled.

The performance of the VES-13 was assessed by investigating the discrimination (equivalent to the area under the receiving operating curve (ROC)). This score ranges from 0 to 1 where a value of 0.5 represents the same performance as chance, 0.5–0.7 represents poor model discrimination, 0.7–0.9 represents reasonable discrimination and ≥0.9 represents excellent discrimination [18]. Discrimination was assessed using the non-parametric method by calculating a c-statistic with 95% confidence intervals for each measure considered as continuous variables.

A series of receiver operating curves (ROC) plots were generated to examine visually the differences in predicting the outcomes of interest. Model goodness of fit was assessed using the Hosmer-Lemeshow statistic. All analyses were conducted using Stata Version 13. (StataCorp, Texas, US) The ‘rocreg‘and ‘rocreg plot’ command was used to generate c-statistics and ROC curves respectively. These commands incorporate bootstrapping in order to obtain the standard error of the c-statistic and the 95% confidence intervals. In addition the sensitivity and specificity of the VES-13 at its high-risk cut-point of ≥3 were calculated for all outcomes of interest.

## Results

### Baseline characteristics

Of a study sample of 904, a total of 862 (95%) study participants had completed the VES-13 at baseline and could be included in the analysis. Of these study participants a total of 326 (38%) were classified as vulnerable at baseline (VES-13 score ≥3). The baseline characteristics of the study participants are presented in Table 1.

**Table 1:** Baseline characteristics of study participants (*n* = 862)

Patient characteristic	Median (IQR)
Age	77 (73, 81)
Deprivation	1.33 (−0.64, 3.04)
	N (%)
Gender	
Male	404 (47)
Female	458 (53)
Marital status^a^	
Married	393 (45)
Separated/Divorced	42 (5)
Widowed	278 (32)
Never married/single	148 (17)
Living arrangements	
Husband/Wife/Partner	383 (44)
Family/Relatives	110 (13)
Live alone	327 (38)
Other	42 (5)
Education^b^	
Basic education	531 (62)
Upper and post-secondary	325 (38)
Social class	
Unskilled	326 (38)
Skilled	536 (62)

^a^Marital status was missing for *n* = 1. ^b^Education was missing for *n* = 6

### Outcome: 1) mortality

**Fig. 1:**
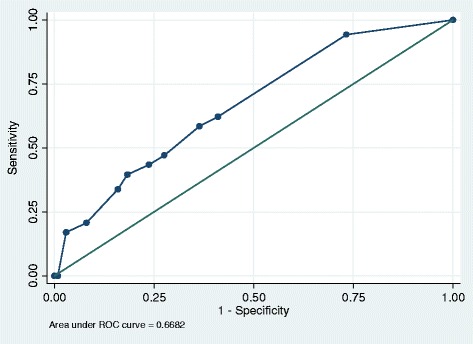
The VES-13 for predicting mortality: receiver operating curve (ROC) plot. This plots the proportion of true positive cases (patients classified as vulnerable who died during the follow-up period i.e. sensitivity) against the proportion of false positives (patients who were not classified as vulnerable yet died during the follow-up period i.e. 1 —specificity) according to changes in the VES-13 cut-points

A total of 53 (6%) study participants died during the study follow-up period. The VES-13 demonstrated relatively poor discrimination (c-statistic: 0.67 (95% CI 0.60, 0.73) for predicting mortality (see Fig. 1). At the cut-point of ≥3 denoting high-risk, the c-statistic was 0.61 (95% CI 0.54, 0.67), with model sensitivity of 59% and specificity of 64% (See Table 2).

**Table 2:** Sensitivity and specificity of the VES-13 for predicting mortality and emergency/ACS admissions at cut-point of ≥3 denoting high-risk (total *n* = 862)

Outcome	Proportion (*n*, %) classifed as high-risk	Proportion (*n*,%) of high-risk group with outcome	Proportion (*n*, %) classifed as low-risk	Proportion (*n*, %) of low-risk group with outcome	Sensitivity VES-13 ≥ 3 (%)	Specificity VES-13 ≥ 3 (%)
Mortality	326 (38%)	31 (9.5%)	536 (62%)	22 (4.1%)	59	64
≥1 Emergency admission	326 (38%)	126 (38.7%)	536 (62%)	120 (22.4%)	51	68
≥1 ACS emergency admission	326 (38%)	67 (20.7%)	536 (62%)	43 (8.1%)	61	66

### Outcome: 2) emergency admission

A total of 246 study participants (29%) were admitted as an emergency at least once during 2 years follow-up. Of these 159 (18%) were admitted once, 56 (7%) were admitted twice and 31 (4%) were admitted ≥3 times. A total of 110 study participants (13%) had an ACS emergency admission.

**Fig. 2:**
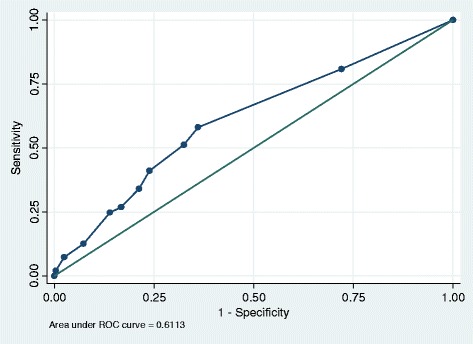
The VES-13 for predicting ≥1 emergency admissions: ROC plot. This plots the proportion of true positive cases (patients classified as vulnerable who had an emergency admission during the follow-up period i.e. sensitivity) against the proportion of false positive cases (patients who were not classified as vulnerable yet were admitted as an emergency i.e. 1 —specificity) according to changes in the VES-13 cut-points

**Fig. 3:**
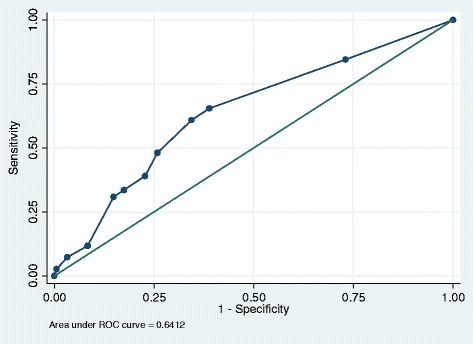
The VES-13 for predicting ≥1 ACS emergency admissions: ROC plot. This plots the proportion of true positive cases (patients classified as vulnerable who had an ACS emergency admission during the follow-up period i.e. sensitivity) against the proportion of false positive cases (patients who are not classified as vulnerable yet had an ACS admission i.e. 1 —specificity) according to changes in the VES-13 cut-points

Overall the VES-13 demonstrated poor performance in predicting ≥1 emergency admission (c-statistic 0.61 (95% CI 0.57, 0.65)) (see Fig. 2) and ≥1 ACS emergency admission (c-statistic 0.64 (95% CI 0.60, 0.68)) (see Fig. 3). At the high-risk cut-point of ≥3, the VES-13 demonstrated poor discrimination for predicting ≥1 emergency admission (c-statistic: 0.59 (95% CI 0.56, 0.63) and ≥1 ACS emergency admission (c-statistic: 0.63 (95% CI 0.60, 0.67) (See Figs. 2 and 3). At this cut-point model sensitivity and specificity were 51% and 68% respectively for ≥1 emergency admission and 61 and 66% respectively for ≥1 ACS emergency admission (See Table 2).

## Discussion

### Principal findings

At baseline, a total of 326 (38%) older people were categorised as vulnerable according to the VES-13 (score ≥3). This is similar to the proportion of older people identified as vulnerable in previous validation studies in the US and Ireland [4, 10]. The VES-13 was not a useful predictor of mortality, emergency admission or ACS admission in this study.

### Comparison with previous literature

While there is increasing interest on the impact of multimorbidity (the presence of ≥2 chronic medical conditions in an individual) on adverse health outcomes, morbidity burden alone is not sufficient to predict mortality and emergency admission [19, 20]. Other predictors captured by the VES-13 such as self-rated health and functional ability are important to consider in predicting poorer health outcomes for older people living in the community. A US study examining the performance of the VES-13 in a cohort of older ambulatory care people (*n* = 649) reported that it successfully predicted functional decline or mortality over 4.5 years of follow up (c-statistic 0.75 (95% CI 0.71–0.80) [4]. The VES-13 has been examined in patients with end-stage cancer diagnoses and in one study (*n* = 197, community palliative care) the VES-13 predicted death within 100 days [21]. In a US case-control study (*n* = 377) of older community-dwelling men with prostate cancer the VES-13 successfully predicted mortality over 5 years of follow up [22]. The current study indicates that model discrimination for mortality was poor but it is important to highlight that the study population which excluded people undergoing palliative care and those with severe cognitive impairment at baseline likely underestimated mortality. In addition, previous studies have examined mortality over longer time periods of up to 5 years whereas the current study had a shorter follow-up period of 2 years [4].

The VES-13 has previously been tested in a cross-sectional Irish study (*n* = 2,033, aged ≥65 years) where it was reported to be associated with increased self-reported healthcare use including primary care visits and inpatient stays.(242) Other measures largely comprising of functional status items have been used to predict future admission with varying results. A US study (*n* = 6,465, aged ≥65 years) examined the value of a different measure of functional status, the Functional Status Indicator (FSI), in predicting future hospital admission.(335) Though reported discrimination was poor, the FSI was as good as two multimorbidity measures (the Charlson comorbidity index and the Chronic Disease Score) in predicting this outcome (c-statistic 0.68 (no CIs reported)).(335) However, this study presented a secondary analysis of data collected for the purposes of a randomised controlled trial (RCT) which is a methodological limitation. A one-year prospective Dutch primary care study (*n* = 430, ≥70 years) tested the performance of three measures of frailty: the Groningen Frailty Indicator; the Tilburg Frailty Indicator; and, the Sherbrooke Postal Questionnaire, in predicting admission [23]. Reported c-statistics for the three measures were poor overall; c-statistic 0.54 (95% CI 0.46, 0.61), 0.60 (95% CI 0.52, 0.67) and 0.60 (95% CI 0.53, 0.67) respectively [23].

The current study builds on this previous research and suggests that the VES-13 is not a useful tool in predicting future emergency admission. This indicates that poorer functioning in older community-dwellers does not, in itself, determine emergency admission risk. However, vulnerability and frailty measures may well have a role when considered in conjunction with other important risk factors in predicting future emergency admission [11].

### Clinical and research implications

An ageing population requires novel and innovative approaches in identifying and managing community-dwelling older people who are more likely to experience poorer health outcomes and require emergency admission. The VES-13 has several practical advantages in that it can be easily administered in approximately 5 minutes and calculating the patient’s score is straightforward. It has, therefore, good clinical utility and has been used by both primary care providers and allied health professionals to help prioritise older people for comprehensive geriatric assessment. The VES-13 performs similarly compared to other available frailty tools validated in primary care populations. For instance, in a prospective primary care study (*n* = 430, aged ≥70 years) which compared three frailty measures, sensitivity ranged from 71 to 83% and specificity from 48 to 63% [23]. An ideal diagnostic test would have high reported sensitivity and specificity, but in reality this is a difficult balance to achieve. In a systematic review of frailty instruments for use in primary care several tools were identified that demonstrated high sensitivity but poor specificity, with the authors’ concluding that these tools should not be used in isolation to predict frailty [24]. Therefore the use of any risk model requires recognition of its limitations and clinical interpretation.

In the current study the VES-13 did not successfully predict emergency admission or mortality. Emergency admission is an inherently difficult outcome to predict [12]. Several risk prediction models have been developed for the purposes of identifying older community-dwelling people at high risk of emergency admission but only a small number achieve good reported predictive accuracy for this outcome [11, 25]. It has been suggested that considering functional status may be important in improving the performance of existing emergency admission risk prediction models [11]. While the VES-13 did not predict emergency admission in the current study, it may be that when combined with other predictors known to be important drivers of future admission, such as prior hospitalisation and multimorbidity, that predictive accuracy improves. Incorporating measures of functional status into existing emergency admission risk models may have a role in improving predictive accuracy. In terms of mortality prediction there has been limited validation of the VES-13 in predicting this outcome outside of North America and in study populations without an underlying cancer diagnosis.

It is important for any risk prediction tool developed in one setting to be tested in new settings of care and populations before widespread use [11]. The VES-13 has been extensively validated in North America and in populations with an index diagnosis of cancer. External validation studies examining the tool in different health settings and healthier populations are needed and an interesting area for future research.

### Strengths and limitations

This prospective primary care study examined the predictive accuracy of the VES-13 for mortality and emergency admission in older community-dwelling people. The study population was not selected based on the presence of any one particular index condition, which improves the generalisability of the findings. The sample size is large compared to previous studies examining this model. The outcomes of emergency admission and ACS admission were recorded from review of the GP medical record. Examining ACS admissions as an outcome of interest is novel. This subset of emergency admissions are becoming increasingly of interest due to perceived preventability through primary care interventions. However, to date only a limited number of risk prediction models have been developed specifically to identify ACS admissions [11]. Understanding the role of vulnerability in predicting this outcome is important and adds to the limited literature in this area.

There are study limitations. This cohort study was originally established to examine the prediction of adverse health outcomes including ADEs, which required participants to complete an interview regarding their medications, and complete a postal questionnaire. As a result, older people undergoing palliative care and those with severe cognitive impairment were excluded. This may have resulted in underestimation of those identified as vulnerable and the mortality outcome. In addition, questionnaire non-respondents were older and had more comorbidity than respondents, which may have resulted in an underestimation of the predictive accuracy of the VES-13. In this study the VES-13 was administered by postal questionnaire whereas in some previous studies it has been administered by interview with trained assessors. However, there are practical advantages to the use of postal questionnaire and this adds to the clinical utility of this tool in practice.

Misclassification bias may be considered an issue but to reduce the risk of this all GP medical record data was recorded by the same researcher and a random sample of 10% of all data was double checked by an independent reviewer with extensive data cleaning undertaken to ensure accuracy. A total of 21 study participants (3%) had some missing data for the outcome measure of functional decline and were excluded. However this proportion of missing data is very small when compared to similar prospective studies [9, 26].

## Conclusion

In this study the VES-13 demonstrated relatively poor discrimination for mortality and emergency admission. External validation studies examining this tool in different health settings and healthier populations are needed and represent an interesting area for future research.

## Additional files

Additional file 1: The Vulnerable Elder’s Survey (VES-13). (DOC 148 kb)
Additional file 2: Ambulatory care sensitive (ACS) emergency admissions. (DOC 36 kb)

## Abbreviations

GP: General practice
VES-13: Vulnerable Eelders Survey
