# Supplementary material for: External validation of the Vulnerable Elder’s Survey for predicting mortality and emergency admission in older community-dwelling people: a prospective cohort study
Source: BMC Geriatr. 2017 Mar 20;17:69. doi: 10.1186/s12877-017-0460-1 (PMC5359866; doi:10.1186/s12877-017-0460-1)

**Additional file 1:**

**-File name** The Vulnerable Elder’s Survey (VES-13)

**-Title of data** The Vulnerable Elder’s Survey (VES-13)

**-Description of data** Questionnaire


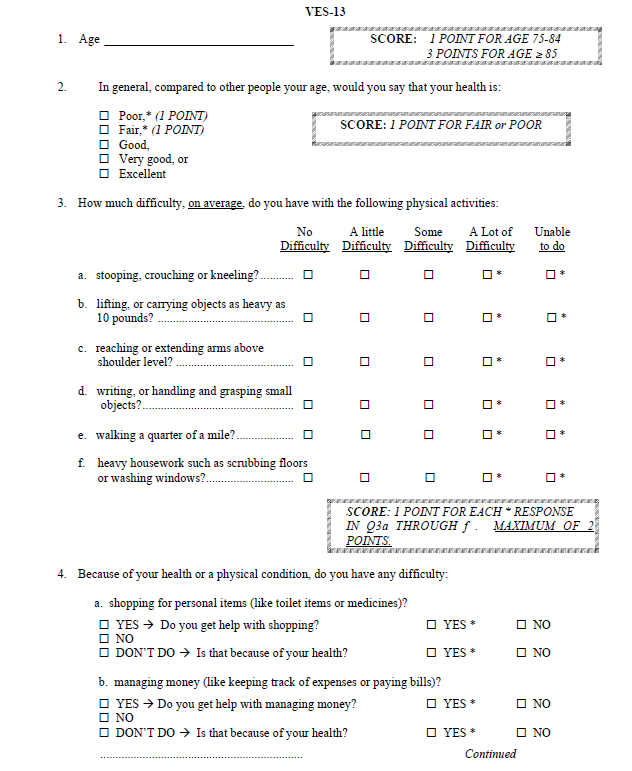


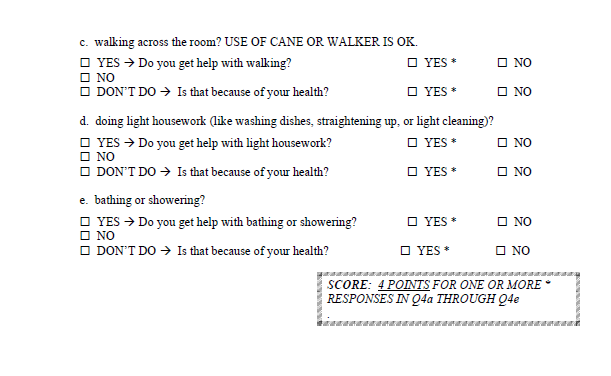

Supplement: Additional file 1: — The Vulnerable Elder’s Survey (VES-13). (DOC 148 kb) [file 12877_2017_460_MOESM1_ESM.doc]
