# Supplementary material for: External validation of the Vulnerable Elder’s Survey for predicting mortality and emergency admission in older community-dwelling people: a prospective cohort study
Source: BMC Geriatr. 2017 Mar 20;17:69. doi: 10.1186/s12877-017-0460-1 (PMC5359866; doi:10.1186/s12877-017-0460-1)
Supplement: Additional file 2: — Ambulatory care sensitive (ACS) emergency admissions. (DOC 36 kb) [file 12877_2017_460_MOESM2_ESM.doc]

**Additional file 2:**

**-File name** Ambulatory care sensitive (ACS) emergency admissions

**-Title of data** Ambulatory care sensitive (ACS) emergency admissions

**-Description of data** List of ambulatory care sensitive (ACS) emergency admissions

Ambulatory care sensitive (ACS) emergency admissions

| **Acute conditions** | **Chronic conditions** | **Other and vaccine- preventable conditions** |
| --- | --- | --- |
| Cellulitis | Angina | Inﬂuenza |
| Dehydration | Asthma | Pneumonia |
| Dental conditions | COPD | Other vaccine-preventable |
| Ear, nose and throat infections | Congestive heart failure |  |
| Gangrene | Convulsions and epilepsy |  |
| Gastroenteritis | Diabetes complications |  |
| Nutritional deﬁciencies | Hypertension |  |
| Pelvic inﬂammatory disease | Iron deﬁciency anaemia |  |
| Perforated/bleeding ulcer |  |  |
| Pyelonephritis |  |  |
